# Does comorbidity explain the ethnic inequalities in cervical cancer survival in New Zealand? A retrospective cohort study

**DOI:** 10.1186/1471-2407-11-132

**Published:** 2011-04-12

**Authors:** Naomi Brewer, Barry Borman, Diana Sarfati, Mona Jeffreys, Steven T Fleming, Soo Cheng, Neil Pearce

**Affiliations:** 1 Centre for Public Health Research, Massey University, PO Box 756, Wellington 6140, New Zealand; 2 Department of Public Health, University of Otago Wellington, PO Box 7373, Wellington 6242, New Zealand; 3 Department of Social Medicine, University of Bristol, Canynge Hall, 39 Whatley Road, Bristol BS8 2PS, UK; 4 Epidemiology, University of Kentucky College of Public Health, 121 Washington Avenue, Lexington, KY 40536-0003, USA; 5 Department of Medical Statistics, Faculty of Epidemiology and Public Health, London School of Hygiene and Tropical Medicine, Keppel Street, London, WC1E 7HT, UK

## Abstract

**Background:**

There are large ethnic differences in cervical cancer survival in New Zealand that are only partly explained by stage at diagnosis. We investigated the association of comorbidity with cervical cancer survival, and whether comorbidity accounted for the previously observed ethnic differences in survival.

**Methods:**

The study involved 1,594 cervical cancer cases registered during 1994-2005. Comorbidity was measured using hospital events data and was classified using the Elixhauser instrument; effects on survival of individual comorbid conditions from the Elixhauser instrument were also assessed. Cox regression was used to estimate adjusted cervical cancer mortality hazard ratios (HRs).

**Results:**

Comorbidity during the year before diagnosis was associated with cervical cancer-specific survival: those with an Elixhauser count of ≥3 (compared with a count of zero) had a HR of 2.17 (1.32-3.56). The HR per unit of Elixhauser count was 1.25 (1.11-1.40). However, adjustment for the Elixhauser instrument made no difference to the mortality HRs for Māori and Asian women (compared to 'Other' women), and made only a trivial difference to that for Pacific women. In contrast, concurrent adjustment for 12 individual comorbid conditions from the Elixhauser instrument reduced the Māori HR from 1.56 (1.19-2.05) to 1.44 (1.09-1.89), *i.e*. a reduction in the excess risk of 21%; and reduced the Pacific HR from 1.95 (1.21-3.13) to 1.62 (0.98-2.68), *i.e*. a reduction in the excess risk of 35%.

**Conclusions:**

Comorbidity is associated with cervical cancer-specific survival in New Zealand, but accounts for only a moderate proportion of the ethnic differences in survival.

## Background

In 2005, cervical cancer was the ninth most common site of cancer registration for New Zealand females [1], and the incidence and mortality rates were moderately high compared with the rest of the developed world [2]. Incidence and mortality rates are not the same across ethnic groups within New Zealand. For example, in 2005, Māori women had an incidence rate of 9.0, Pacific women 16.3 and 'Other' (predominantly European) women 5.6 per 100,000 women; Māori women had a mortality rate of 6.5, Pacific women 7.1 and 'Other' women 1.4 per 100,000 women [1].

We have previously reported demographic differences in cervical cancer survival in New Zealand [3]. Māori and Pacific women had higher death rates than 'Other' women, whereas Asian women had a lower risk. Adjustment for stage at diagnosis, socio-economic position (SEP), and urban/rural residence explained only some of the increased risks in Māori and Pacific women. Ethnic differences in stage at diagnosis were not entirely explained by differences in screening history [4,5]. There is some evidence of limited differences in treatment between Māori and non-Māori women, but these differences have little impact on survival differences [6]. Thus, the reasons for the differences in survival are currently unclear, but one possibility not previously examined is that they may, in part, be due to differences in comorbidity at the time of cervical cancer diagnosis. Māori and Pacific women have higher rates of many diseases, including smoking-related respiratory diseases, diabetes and cardiovascular disease [7-9]. Such comorbid conditions may have effects prior to diagnosis (*e.g*. influence the likelihood of cancer screening or late-stage diagnosis) [10,11], or affect survival post-diagnosis either directly (*e.g*. some comorbid conditions may adversely affect prognosis) or indirectly (*e.g*. some comorbid conditions may affect or limit treatment options or decisions). In New Zealand, comorbidity has been found to contribute to ethnic-specific survival disparities for colon cancer [12], the management of stages I and II non-small-cell lung cancer [13], and adverse event status, inpatient death and increased length of stay in selected Auckland hospitals [14]. Internationally comorbidity has also been found to adversely affect survival in patients with a range of conditions, including cervical cancer [15-21].

We therefore investigated the associations of various comorbid conditions with cervical cancer survival, and whether these comorbid conditions accounted for the previously observed ethnic differences in survival.

## Methods

The source population comprised all cervical cancer cases registered with the New Zealand Cancer Registry (NZCR) between 1 January 1994 and 30 December 2005 [3,4]. The NZCR records self-identified ethnicity, and allows for multiple responses. Participants who reported more than one ethnicity were classified into a single ethnicity using the standard system of prioritisation: Māori > Pacific > Asian > 'Other' [22]. Participants with missing ethnicity data were included in the 'Other' (predominantly European) ethnic group in the analyses. This approach is standard practice in New Zealand health research [23,24]. All registrations include the National Health Index (NHI) number which uniquely identifies individual health care users; this was used to obtain cause-specific mortality data (from the Mortality Collection) up to the end of December 2005 (the most recent year for which data was available), and hospital events data (from the National Minimum Dataset (NMDS); up to 99 diagnosis/procedure codes may be provided to the NMDS) from 1988 to 31 December 2005.

SEP was estimated using the New Zealand Deprivation Index 2001 (NZDep), an area-based measure derived from a combination of nine socioeconomic variables derived from the national census [25]. Each participant was assigned a score based upon the residential area (the domicile code) in which they lived, as recorded on the NZCR at the time of registration. These scores were then grouped into quintiles [25].

The domicile code recorded for each participant was also used to assign urban/rural residence according to population size [26]. Participants were classified as living in a main urban area (with a population of ≥30,000), a secondary or minor urban area (population ≥1,000 to 29,999), or a rural area (population <1,000).

Data on stage at diagnosis were obtained from the NZCR, and reported using the International Federation of Gynecology and Obstetrics (FIGO) system [27]. In order to provide sufficient numbers in each category, the FIGO stages were grouped into four categories: stages 0 to IB2; II to IIB; III to IIIB; IVA to IVB. A fifth category of 'missing' was utilised for cases where the FIGO stage was unknown. We conducted a basic sensitivity analysis, to assess the potential for bias resulting from the exclusion of the women with missing stage data; this involved three sets of analyses: (i) adjusting for stage and excluding the women with missing stage data; (ii) including the women with and without missing stage data, and adjusting for stage with a dummy variable representing the women with missing stage data; and (iii) including the women with and without missing stage data, but not adjusting for stage. The three sets of analyses yielded the same patterns. We therefore present here the findings from the first method (i.e. excluding women with missing stage), because it is necessary to adjust for stage, and this is the only approach that enables us to do this validly [3,4].

We used two widely utilised comorbidity measures. The Elixhauser instrument [28] was designed specifically for use with administrative data, and is based on a set of 30 comorbid conditions which were associated with increased length of stay, hospital charges and mortality among non-maternal inpatients in California in 1992 [28]. The Charlson Comorbidity Index (CCI) [29] comprises 19 comorbid conditions which are given a weight of 1 to 6 on the basis of the strength of their association with one-year mortality among a cohort of 607 general medical patients in the United States [29]. To our knowledge this is the first study of the role of comorbidity in cervical cancer survival in New Zealand, and there was therefore no prior data on which of these two (or any other) comorbidity measures were most appropriate to use. In general, we found very similar results with the two comorbidity measures, and we have therefore only reported the findings for the Elixhauser instrument (the findings for the CCI are available as Additional file 1 Tables S1-S4); effects on survival of individual comorbid conditions from the Elixhauser instrument were also assessed.

Comorbidity was assessed, using the hospital events data, according to the enhanced ICD-9-CM (for data from 1988-1999) and ICD-10 (for data from 2000-2005) coding algorithms of Quan *et al *[30] for the Elixhauser instrument [28] and the CCI [29]. We used both the primary and the secondary diagnoses fields to identify comorbid conditions during the period one year, and the period five years, preceding, and including, the date of diagnosis. The optimal look-back period (the time over which to identify comorbid conditions) was not clear since shorter times may capture more active conditions and longer periods may be more likely to identify all of the important comorbid conditions [31]. We therefore utilised two look-back periods, with five years being the longest timeframe over which we had data for all of the women. In general, we found similar results with the two look-back periods (see Additional file 1 Tables S1-S3), though the associations of comorbidity with survival were somewhat stronger when using the one-year look-back period; we therefore only report here the findings for the one-year look-back period. We included comorbid conditions identified up to and including the date of diagnosis to strike a balance between identifying all of the comorbid conditions that the women had at the time of diagnosis whilst attempting to avoid including conditions that may have been caused by treatment after diagnosis. Metastatic solid tumours were excluded from both comorbidity algorithms, as were all diagnosis codes for cervical cancer. For each woman, the comorbidity frequency (for the Elixhauser instrument) and score (for the CCI) were recorded (for use as continuous variables) and were also then categorised into (two sets of) four groups (0, 1, 2, and ≥3).

The Elixhauser measure was calculated using the Statistical Analysis System (SAS) software 9.1, whilst all other analyses were conducted using Intercooled Stata 10 for Windows (StataCorp, College Station, Texas, USA). The Cox proportional hazards model [32] was used to estimate the hazard ratios (HRs) for cervical cancer mortality, 'other mortality' (non-cervical cancer), and total mortality associated with the Elixhauser count, as well as with ethnicity, NZDep, and urban/rural residence, adjusted for age, registration year, and stage at diagnosis. Women were censored at the time of their death or on 31 December 2005 if they were still alive at that time [3]. In the final set of analyses, the HRs for each ethnic group were estimated adjusted for the Elixhauser count, and, finally, for the individual comorbid conditions that had a HR of ≥1.5.

The New Zealand Central Ethics Committee granted ethical approval for the study (CEN/08/04/EXP).

## Results

There were 2,323 cases of cervical cancer registered on the NZCR between 1 January 1994 and 31 December 2005, and all of these cases were included in the descriptive analyses of comorbidity (Table 1). Using a one-year look-back period, 15.6% of cases had had at least one comorbidity (included in the Elixhauser instrument) event in the year before diagnosis; the percentages were similar in Asian (13.3%) and 'Other' women (13.7%), but were highest in Pacific women (32.4%), with Māori women having an intermediate value (19.7%). The Elixhauser count was strongly associated with NZDep, and FIGO stage, but was only weakly associated with urban/rural residence and time period of diagnosis.

**Table 1:** Characteristics of cervical cancer cases, n (%)

		Elixhauser co-morbidities			
		
	Total	0	1	2	3+
**Total**	2,323 (100)	1,960 (84.4)	223 (9.6)	63 (2.7)	77 (3.3)
**FIGO stage**					
0 to IB2	1,155 (49.7)	1,067 (92.4)	63 (5.5)	13 (1.1)	12 (1.0)*
II to IIB	262 (11.3)	207 (79.0)	32 (12.2)	11 (4.2)	12 (4.6)
III to IIIB	232 (10.0)	169 (72.8)	41 (17.7)	16 (6.9)	6 (2.6)
IVA to IVB	53 (2.3)	33 (62.3)	11 (20.8)	3 (5.7)	6 (11.3)
Missing	621 (26.7)	484 (77.9)	76 (12.2)	20 (3.2)	41 (6.6)
**Ethnicity**					
Other	1,674 (72.1)	1,444 (86.3)	141 (8.4)	41 (2.5)	48 (2.9)*
Māori	416 (17.9)	334 (80.3)	50 (12.0)	15 (3.6)	17 (4.1)
Pacific	105 (4.5)	71 (67.6)	21 (20.0)	3 (2.9)	10 (9.5)
Asian	128 (5.5)	111 (86.7)	11 (8.6)	4 (3.1)	2 (1.6)
**NZDep2001, quintiles**					
1 (Least deprived)	298 (12.8)	277 (93.0)	14 (4.7)	3 (1.0)	4 (1.3)**
2	333 (14.3)	283 (85.0)	33 (9.9)	8 (2.4)	9 (2.7)
3	416 (17.9)	350 (84.1)	37 (8.9)	12 (2.9)	17 (4.1)
4	526 (22.6)	432 (82.1)	56 (10.7)	19 (3.6)	19 (3.6)
5 (Most deprived)	623 (26.8)	510 (81.9)	67 (10.8)	21 (3.4)	25 (4.0)
Missing	127 (5.5)	108 (85.0)	16 (12.6)	0	3 (2.4)
**Urban/rural residency**					
Main urban	1,640 (70.6)	1,403 (85.6)	141 (8.6)	42 (2.6)	54 (3.3)^NS^
Secondary urban	361 (15.5)	288 (79.8)	47 (13.0)	13 (3.6)	13 (3.6)
Rural	196 (8.4)	162 (82.7)	19 (9.7)	8 (4.1)	7 (3.6)
Missing	126 (5.4)	107 (84.9)	16 (12.7)	0	3 (2.4)
**Year of diagnosis**					
1994-1997	843 (36.3)	714 (84.7)	82 (9.7)	24 (2.9)	23 (2.7)^NS^
1998-2001	815 (35.1)	689 (84.5)	79 (9.7)	20 (2.5)	27 (3.3)
2002-2005	665 (28.6)	557 (83.8)	62 (9.3)	19 (2.9)	27 (4.1)

p value based on Pearson's chi-squared

* p = 0.0001

** p = 0.02

NS: Not significant at 95%

For the analyses of the effects of comorbidity on mortality (Table 2), the following exclusions were made; 621 because they did not have a FIGO code (including 17 women whose cancer registration was made on the date of the their death, and 50 women that could not be assigned an NZDep score), 77 cases because they did not have a domicile code that could be assigned an NZDep score, and a further 31 cases because they were diagnosed after 30 June 2005 (and therefore had a potential follow-up time of less than six months), leaving 1,594 women to be included in the analyses. The women that were excluded because they did not have a FIGO code had a similar ethnic and SEP distribution to the cases that did have a FIGO code [4].

**Table 2:** Mortality by comorbidity

Comorbidity	HR (95%CI)^a^
**Cervical cancer**	
Elixhauser (1 unit)	1.25 (1.11-1.40)
Elixhauser 0	1.00^b^
Elixhauser 1	1.29 (0.96-1.75)
Elixhauser 2	1.33 (0.83-2.13)
Elixhauser 3+	2.17 (1.32-3.56)
**Other mortality (not cervical cancer)**	
Elixhauser (1 unit)	1.46 (1.18-1.79)
Elixhauser 0	1.00^b^
Elixhauser 1	2.49 (1.39-4.44)
Elixhauser 2	2.62 (1.20-5.72)
Elixhauser 3+	2.76 (1.04-7.30)
**Total mortality**	
Elixhauser (1 unit)	1.28 (1.15-1.41)
Elixhauser 0	1.00^b^
Elixhauser 1	1.47 (1.13-1.92)
Elixhauser 2	1.48 (0.99-2.21)
Elixhauser 3+	2.20 (1.41-3.41)

^a ^Adjusted for age, year of diagnosis, stage, ethnicity, socioeconomic position, and urban/rural residence

^b ^Reference category

Of the 1,594 women included in the analyses: 99.2% were diagnosed based upon the histology of the primary malignant tumour [3]; 1,163 (73%) identified as 'Other' ethnicity, 312 of whom died during the follow-up period, 241 (77%) due to cervical cancer, and 71 (23%) due to other causes; 292 identified as Māori ethnicity (18%), 104 of whom died, 92 (88%) due to cervical cancer, and 12 (12%) due to other causes; 59 (4%) identified as Pacific ethnicity, 20 of whom died, 20 (100%) due to cervical cancer; and, 80 (5%) identified as Asian ethnicity, 14 of whom died, 13 (93%) due to cervical cancer, and 1 (7%) due to other causes.

Table 2 shows the HRs for cervical cancer survival by comorbidity, adjusted for age, year of diagnosis, stage, ethnicity, NZDep, and urban/rural residence. Comorbid disease in the year before diagnosis was associated with cervical cancer-specific survival: those with an Elixhauser count of 3 or more had a HR of 2.17 (1.32-3.56). The HR was associated with a per unit increase (when analysing the Elixhauser instrument as a continuous variable) of 1.25 (1.11-1.40). Comorbidity was more strongly associated with mortality from conditions other than cervical cancer: those with an Elixhauser count of 3 or more had a HR for other mortality of 2.76 (1.04-7.30). The HR was associated with a per unit increase of 1.46 (1.18-1.79).

We estimated the cervical cancer-specific survival HRs adjusted for age, year of diagnosis, stage, ethnicity, NZDep and urban/rural residence, for those with individual conditions included in the Elixhauser instrument (see Additional file 1 Table S3). Thirteen of the individual comorbid conditions showed HRs of ≥1.5 in the one-year look-back period; congestive heart failure (2.35 95% CI 1.22-4.52), valvular disease (2.84, 0.70-11.61), complicated hypertension (1.74, 0.24-12.72), chronic pulmonary disease (1.62, 0.95-2.77), uncomplicated diabetes (2.17, 1.33-3.53), complicated diabetes (10.46, 3.01-36.37), renal failure (4.27, 2.08-8.76), liver disease (2.43, 0.76-7.78), coagulopathy (2.78, 0.68-11.43), obesity (3.52, 1.55-7.98), fluid and electrolyte disorders (4.03, 2.01-8.08), blood loss anaemia (2.44, 1.48-4.00), and drug abuse (3.28, 0.45-23.76). We therefore adjusted for these individual comorbid conditions in the final analyses, except for uncomplicated diabetes because the methodology of the Elixhauser instrument allows for a woman to be recorded as having both uncomplicated and complicated diabetes (where the Elixhauser count was used, only complicated diabetes (or complicated hypertension) was included when the woman also had uncomplicated diabetes (or uncomplicated hypertension)).

Table 3 shows the findings for ethnic differences in cervical cancer-specific survival adjusted for comorbidity as a continuous variable and for the 12 individual comorbid conditions. Adjustment for the Elixhauser count made no difference to the cervical cancer-specific survival HRs for Māori and Asian women (compared to 'Other' women), and made only a trivial difference to that for Pacific women. The largest change was for Pacific women, where the HR fell from 1.95 (1.21-3.13) to 1.92 (1.20-3.09). However, the HRs changed more substantially when adjustment was made for all 12 of the individual comorbid conditions; the HR for Māori women fell from 1.56 (1.19-2.05) to 1.44 (1.09-1.89), representing a 21% decrease in the excess mortality risk; the HR for Pacific women fell from 1.95 (1.21-3.13) to 1.62 (0.98-2.68), representing a 35% decrease in the excess mortality risk.

**Table 3:** Cervical cancer-specific mortality by ethnicity adjusted for comorbidity

		Ethnicity			
Comorbidities	Comorbidity	Other	Māori	Pacific	Asian
	**HR (95%CI)**^a^	**HR (95%CI)^b^**	**HR (95%CI)^c^**	**HR (95%CI)^c^**	**HR (95%CI)^c^**
No comorbidity adjustment/inclusion		1.00	1.56 (1.19-2.05)	1.95 (1.21-3.13)	0.72 (0.41-1.27)
Elixhauser as continuous variable	1.25 (1.11-1.40)	1.00	1.55 (1.19-2.04)	1.92 (1.20-3.09)	0.72 (0.41-1.26)
Individual comorbities					
Congestive heart failure	2.35 (1.22-4.52)	1.00	1.57 (1.20-2.06)	1.98 (1.23-3.17)	0.72 (0.41-1.27)
Valvular disease	2.84 (0.70-11.61)	1.00	1.56 (1.19-2.04)	1.96 (1.22-3.14)	0.72 (0.41-1.27)
Hypertension, complicated	1.74 (0.24-12.72)	1.00	1.57 (1.19-2.06)	1.95 (1.22-3.13)	0.72 (0.41-1.27)
Chronic pulmonary disease	1.62 (0.95-2.77)	1.00	1.55 (1.18-2.03)	1.95 (1.22-3.13)	0.67 (0.38-1.19)
Diabetes, complicated	10.46 (3.01-36.37)	1.00	1.55 (1.18-2.04)	1.70 (1.03-2.80)	0.71 (0.40-1.25)
Renal failure	4.27 (2.08-8.76)	1.00	1.58 (1.20-2.07)	1.70 (1.04-2.77)	0.72 (0.41-1.27)
Liver disease	2.43 (0.76-7.78)	1.00	1.55 (1.18-2.03)	1.92 (1.20-3.09)	0.72 (0.41-1.26)
Coagulopathy	2.78 (0.68-11.43)	1.00	1.55 (1.18-2.03)	1.91 (1.19-3.07)	0.72 (0.41-1.27)
Obesity	3.52 (1.55-7.98)	1.00	1.55 (1.18-2.04)	1.90 (1.18-3.05)	0.72 (0.41-1.27)
Fluid and electrolyte disorders	4.03 (2.01-8.08)	1.00	1.51 (1.15-1.98)	1.97 (1.23-3.16)	0.69 (0.39-1.21)
Blood loss anaemia	2.44 (1.48-4.00)	1.00	1.53 (1.17-2.01)	1.98 (1.23-3.17)	0.71 (0.40-1.26)
Drug abuse	3.28 (0.45-23.76)	1.00	1.56 (1.19-2.04)	1.95 (1.22-3.13)	0.72 (0.41-1.27)
Multivariate - all 12 of the above		1.00	1.44 (1.09-1.89)	1.62 (0.98-2.68)	0.63 (0.35-1.13)

^a ^Adjusted for age, year of diagnosis, stage, ethnicity, socioeconomic position, and urban/rural residence

^b ^Reference category

^c ^Adjusted for age, year of diagnosis, stage, ethnicity, socioeconomic position, urban/rural residence, and comorbidity index

## Discussion

This study found that comorbidity is associated with cervical cancer-specific mortality and more strongly with mortality from other causes. This latter finding is not surprising since some cervical cancer patients who have a comorbidity may die from this comorbidity, and this group would therefore be expected to have a higher death rate from "other causes" (i.e. all causes of death other than cervical cancer) than cervical cancer patients who do not have a comorbidity.

Adjusting for the Elixhauser instrument produced little change in the ethnic differences in mortality. In contrast, adjustment for 12 individual comorbid conditions included in the Elixhauser instrument reduced the excess HR for Māori women by 21% and for Pacific women by 35%.

A strength of the study is that the Cancer Registry Act came into effect in 1994 making cancer registration mandatory [1], and case under-ascertainment unlikely [23]. Death registration is also mandatory in New Zealand, and can be linked to cancer registrations using the NHI number; thus there is a high probability that the study identified all of the cases that died in New Zealand. There may have been some misclassification of cause of death, but it is unlikely to have produced significant bias in the ethnic comparisons [33]. Furthermore, classification of the cause of death for patients on the NZCR is highly accurate since in cases that are registered prior to death, information from the Cancer Registry is used to classify the underlying cause of death [34].

Other possible limitations of the study include the potential misclassification of ethnicity, which has been estimated to produce a 17% undercount of Māori cancer registrations [35] (this involves misclassification of ethnicity on registrations, rather than case under-ascertainment). Thus, the 'Other' ethnic group may contain some Māori cases that were incorrectly classified, thereby diluting the ethnic survival differences. There is also evidence of a 6-7% undercount of Māori deaths [35,36], but this would not bias the current study since the ethnicity recorded on the Cancer Registry was used in all analyses. The classification of ethnicity was based on the wording of the corresponding census questions, and these have changed over time, but once again this is unlikely to have produced serious bias because the ethnicity recorded on the Cancer Registry was also used to classify the corresponding deaths, and the analyses were adjusted for year of diagnosis. There may also be misclassification of area-based SEP and urban/rural residence in cancer registrations, but in each instance, any such misclassification is unlikely to be associated with subsequent survival and, if anything, is likely to produce underestimates of the differences in survival between these various demographic groups.

The greatest change in the ethnic-specific HRs occurred when adjustment was made for the 12 individual comorbid conditions, rather than using the summary Elixhauser comorbidity measure. Some of these individual comorbid conditions may have shown elevated HRs by chance, because of the large number of comparisons involved.

The comorbidity data was based on administrative in-hospital data and therefore some conditions may not have been recorded. However, a study on colon cancer in New Zealand found that despite comorbid conditions being recorded more frequently in patients' medical notes than in administrative data, the use of a comorbidity measure still improved the prediction of all-cause survival in a multivariable model [37]. It is also possible that some patients had undiagnosed disease, but misclassification of this type would probably decrease the effect of comorbidity on survival [37].

To date, there have been few studies of the role of comorbid conditions in cervical cancer survival, and none in New Zealand. Our results are generally consistent with those of Coker et al. [15] who found that in Texan women aged 65 years or older with cervical cancer, those that had one or more comorbid conditions were 40% more likely to die (from all causes) compared with women who did not have any comorbid conditions. However, unlike Coker et al. [15] who did not find an independent association between comorbidity and cervical cancer-specific survival, we found an independent 25% increased risk of death from cervical cancer for each unit increase of the Elixhauser count (Table 3). In a study of stage IB squamous cell carcinoma, Hopkins and Morley [17] found that women with diabetes had an 82% cumulative 5-year all-cause survival compared with an 89% survival in those who did not have diabetes (p = 0.04). These findings are also consistent with the 10-fold increased risk of cervical cancer-specific mortality in the present study. In contrast to our study, Leath et al. [38] did not find comorbid conditions to be an independent predictor of survival in women with either early or late stage cervical cancer.

The present study has shown that Māori and Pacific women have a larger number of comorbid conditions than 'Other' and Asian women when measured with the Elixhauser instrument with a one-year look-back period (Table 1). Women living in more deprived areas had larger numbers of comorbid conditions according to the Elixhauser instrument. We found independent associations between the Elixhauser count and cervical cancer-specific, 'other' and total mortality (Table 2).

Reducing ethnic inequalities in cancer is one of the overall purposes of the New Zealand Cancer Control Strategy [39]. We and others [1,3,40,41] have previously demonstrated substantial ethnic inequalities in cervical cancer incidence, mortality and survival in New Zealand. It has been suggested [42] that comorbid conditions, which are known to differ between ethnic groups [7], could account for these inequalities and, as mentioned earlier, there is some international evidence of comorbidity adversely affecting cervical cancer survival [15,17,18]. The current study, the first to empirically investigate this issue in New Zealand, only partially supports this hypothesis. It is possible that there are small ethnic differences at each stage of the cancer continuum (screening, diagnosis, treatment, comorbidity, follow-up, etc) and that each of these makes a small contribution to the major overall ethnic differences in survival that we have reported.

## Conclusion

In summary, we assessed the roles of comorbid conditions identified through hospital events data and found that these conditions are associated with cervical cancer-specific mortality, but account for only a moderate proportion of the ethnic differences in survival. Other factors, including possible differences in treatment and follow-up, may also play a role.

## Competing interests

The authors declare that they have no competing interests.

## Authors' contributions

NB co-initiated and co-led the study design, collected the data, co-led the data analysis and interpretation, wrote the first draft of the paper, co-ordinated draft revisions and wrote the final manuscript. BB contributed to the study design, data analysis and interpretation, and draft revisions. DS contributed to the study design, data analysis and interpretation, and draft revisions. MJ contributed to the study conception and design, data analysis and interpretation, and draft revisions. STF contributed to the study design, data analysis and interpretation, and draft revisions. SC contributed to the data analysis and interpretation, and draft revisions. NP co-initiated and co-led the study design, co-led the data analysis and interpretation, and contributed to draft revisions. All authors read and approved the final manuscript.

## Pre-publication history

The pre-publication history for this paper can be accessed here:

http://www.biomedcentral.com/1471-2407/11/132/prepub

## Supplementary Material

Additional file 1 **Results for the Charlson Comorbidity Index and Elixhauser instrument with both the one-year and the five-year look-back periods**. Table S1 Characteristics of cervical cancer cases; Table S2 Mortality by comorbidity measures; Table S3 Elixhauser comorbid conditions frequency and cervical cancer-specific mortality adjusted for individual comorbid conditions; Table S4 Cervical cancer-specific mortality by ethnicity adjusted for comorbidity with one-year look-back period. Click here for file
