# Supplementary material for: Does comorbidity explain the ethnic inequalities in cervical cancer survival in New Zealand? A retrospective cohort study
Source: BMC Cancer. 2011 Apr 12;11:132. doi: 10.1186/1471-2407-11-132 (PMC3087712; doi:10.1186/1471-2407-11-132)
Supplement: Additional file 1 — Results for the Charlson Comorbidity Index and Elixhauser instrument with both the one-year and the five-year look-back periods. Table S1 Characteristics of cervical cancer cases; Table S2 Mortality by comorbidity measures; Table S3 Elixhauser comorbid conditions frequency and cervical cancer-specific mortality adjusted for individual comorbid conditions; Table S4 Cervical cancer-specific mortality by ethnicity adjusted for comorbidity with one-year look-back period. [file 1471-2407-11-132-S1.DOC]

**Table S**1: Characteristics of cervical cancer cases, n (%)

|  | **Total** | **One-year look-back period** | | | | | | | | **Five-year look-back period** | | | | | | | |  |
| --- | --- | --- | --- | --- | --- | --- | --- | --- | --- | --- | --- | --- | --- | --- | --- | --- | --- | --- |
|  |  | **Charlson Index** | | | | **Elixhauser comorbid conditions** | | | | **Charlson Index** | | | | **Elixhauser comorbid conditions** | | | |  |
|  |  | **0** | **1** | **2** | **3+** | **0** | **1** | **2** | **3+** | **0** | **1** | **2** | **3+** | **0** | **1** | **2** | **3+** |  |
| **Total** | 2,323 (100) | 2,077 (89.4) | 105 (4.5) | 94 (4.1) | 47 (2.0) | 1,960 (84.4) | 223 (9.6) | 63 (2.7) | 77 (3.3) | 1,962 (84.5) | 158 (6.8) | 118 (5.1) | 85 (3.7) | 1,805 (77.7) | 292 (12.6) | 107 (4.6) | 119 (5.1) |  |
| **FIGO stage** | |  |  |  |  |  |  |  |  |  |  |  |  |  |  |  |  |  |
| 0 to IB2 | 1,155 (49.7) | 1,101 (95.3) | 30 (2.6) | 17 (1.5) | 7 (0.6)^$^ | 1,067 (92.4) | 63 (5.5) | 13 (1.1) | 12 (1.0)^$^ | 1,060 (91.8) | 52 (4.5) | 27 (2.3) | 16 (1.4)^$^ | 1,000 (86.6) | 96 (8.3) | 37 (3.2) | 22 (1.9)^$^ |  |
| II to IIB | 262 (11.3) | 228 (87.0) | 9 (3.4) | 21 (8.0) | 4 (1.5) | 207 (79.0) | 32 (12.2) | 11 (4.2) | 12 (4.6) | 208 (79.4) | 18 (6.9) | 24 (9.2) | 12 (4.6) | 182 (69.5) | 46 (17.6) | 14 (5.3) | 20 (7.6) |  |
| III to IIIB | 232 (10.0) | 191 (82.3) | 22 (9.5) | 15 (6.5) | 4 (1.7) | 169 (72.8) | 41 (17.7) | 16 (6.9) | 6 (2.6) | 177 (76.3) | 29 (12.5) | 18 (7.8) | 8 (3.5) | 154 (66.4) | 47 (20.3) | 18 (7.8) | 13 (5.6) |  |
| IVA to IVB | 53 (2.3) | 38 (71.7) | 5 (9.4) | 4 (7.6) | 6 (11.3) | 33 (62.3) | 11 (20.8) | 3 (5.7) | 6 (11.3) | 36 (67.9) | 7 (13.2) | 4 (7.6) | 6 (11.3) | 30 (56.6) | 12 (22.6) | 5 (9.4) | 6 (11.3) |  |
| Missing | 621 (26.7) | 519 (83.6) | 39 (6.3) | 37 (6.0) | 26 (4.2) | 484 (77.9) | 76 (12.2) | 20 (3.2) | 41 (6.6) | 481 (77.5) | 52 (8.4) | 45 (7.3) | 43 (6.9) | 439 (70.7) | 91 (14.7) | 33 (5.3) | 58 (9.3) |  |
| **Ethnicity** |  |  |  |  |  |  |  |  |  |  |  |  |  |  |  |  |  |  |
| Other | 1,674 (72.1) | 1,513 (90.4) | 69 (4.1) | 67 (4.0) | 25 (1.5)^$^ | 1,444 (86.3) | 141 (8.4) | 41 (2.5) | 48 (2.9)^$^ | 1,427 (85.2) | 109 (6.5) | 84 (5.0) | 54 (3.2)^§^ | 1,325 (79.2) | 190 (11.4) | 77 (4.6) | 82 (4.9)* |  |
| Māori | 416 (17.9) | 363 (87.3) | 22 (5.3) | 20 (4.8) | 11 (2.6) | 334 (80.3) | 50 (12.0) | 15 (3.6) | 17 (4.1) | 341 (82.0) | 32 (7.7) | 25 (6.0) | 18 (4.3) | 308 (74.0) | 64 (15.4) | 20 (4.8) | 24 (5.8) |  |
| Pacific | 105 (4.5) | 82 (78.1) | 9 (8.6) | 7 (6.7) | 7 (6.7) | 71 (67.6) | 21 (20.0) | 3 (2.9) | 10 (9.5) | 78 (74.3) | 10 (9.5) | 9 (8.6) | 8 (7.6) | 66 (62.9) | 23 (21.9) | 5 (4.8) | 11 (10.5) |  |
| Asian | 128 (5.5) | 119 (93.0) | 5 (3.9) | 0 | 4 (3.1) | 111 (86.7) | 11 (8.6) | 4 (3.1) | 2 (1.6) | 116 (90.6) | 7 (5.5) | 0 | 5 (3.9) | 106 (82.8) | 15 (11.7) | 5 (3.9) | 2 (1.6) |  |
| **NZDep, quintiles** | | |  |  |  |  |  |  |  |  |  |  |  |  |  |  |  |  |
| 1 (Least deprived) | 298 (12.8) | 283 (95.0) | 6 (2.0) | 7 (2.4) | 2 (0.7)^NS^ | 277 (93.0) | 14 (4.7) | 3 (1.0) | 4 (1.3)^§^ | 269 (90.3) | 13 (4.4) | 10 (3.4) | 6 (2.0)^NS^ | 261 (87.6) | 19 (6.4) | 11 (3.7) | 7 (2.4)^‡^ |  |
| 2 | 333 (14.3) | 294 (88.3) | 18 (5.4) | 14 (4.2) | 7 (2.1) | 283 (85.0) | 33 (9.9) | 8 (2.4) | 9 (2.7) | 282 (84.7) | 22 (6.6) | 17 (5.1) | 12 (3.6) | 266 (79.9) | 41 (12.3) | 12 (3.6) | 14 (4.2) |  |
| 3 | 416 (17.9) | 369 (88.3) | 22 (5.3) | 14 (3.4) | 11 (2.6) | 350 (84.1) | 37 (8.9) | 12 (2.9) | 17 (4.1) | 349 (83.9) | 30 (7.2) | 17 (4.1) | 20 (4.8) | 325 (78.1) | 43 (10.3) | 21 (5.1) | 27 (6.5) |  |
| 4 | 526 (22.6) | 459 (87.3) | 29 (5.5) | 30 (5.7) | 8 (1.5) | 432 (82.1) | 56 (10.7) | 19 (3.6) | 19 (3.6) | 430 (81.8) | 43 (8.2) | 32 (6.1) | 21 (4.0) | 391 (74.3) | 78 (14.8) | 28 (5.3) | 29 (5.5) |  |
| 5 (Most deprived) | 623 (26.8) | 559 (89.7) | 22 (3.5) | 24 (3.9) | 18 (2.9) | 510 (81.9) | 67 (10.8) | 21 (3.4) | 25 (4.0) | 525 (84.3) | 41 (6.6) | 34 (5.5) | 23 (3.7) | 463 (74.3) | 92 (14.8) | 31 (5.0) | 37 (5.9) |  |
| Missing | 127 (5.5) | 113 (89.0) | 8 (6.3) | 5 (3.9) | 1 (0.8) | 108 (85.0) | 16 (12.6) | 0 | 3 (2.4) | 107 (84.3) | 9 (7.1) | 8 (6.3) | 3 (2.4) | 99 (78.0) | 19 (15.0) | 4 (3.2) | 5 (3.9) |  |
| **Urban/rural residence** | | |  |  |  |  |  |  |  |  |  |  |  |  |  |  |  |  |
| Main urban | 1,640 (70.6) | 1,488 (90.7) | 62 (3.8) | 54 (3.3) | 36 (2.2)** | 1,403 (85.6) | 141 (8.6) | 42 (2.6) | 54 (3.3)^NS^ | 1,405 (85.7) | 104 (6.3) | 76 (4.6) | 55 (3.4)^NS^ | 1,295 (79.0) | 187 (11.4) | 75 (4.6) | 83 (5.1)^NS^ |  |
| Secondary urban | 361 (15.5) | 306 (84.8) | 20 (5.5) | 26 (7.2) | 9 (2.5) | 288 (79.8) | 47 (13.0) | 13 (3.6) | 13 (3.6) | 288 (79.8) | 28 (7.8) | 25 (6.9) | 20 (5.5) | 262 (72.6) | 63 (17.5) | 17 (4.7) | 19 (5.3) |  |
| Rural | 196 (8.4) | 171 (87.2) | 15 (7.7) | 9 (4.6) | 1 (0.5) | 162 (82.7) | 19 (9.7) | 8 (4.1) | 7 (3.6) | 163 (83.2) | 17 (8.7) | 9 (4.6) | 7 (3.6) | 150 (76.5) | 23 (11.7) | 11 (5.6) | 12 (6.1) |  |
| Missing | 126 (5.4) | 112 (88.9) | 8 (6.4) | 5 (4.0) | 1 (0.8) | 107 (84.9) | 16 (12.7) | 0 | 3 (2.4) | 106 (84.1) | 9 (7.1) | 8 (6.4) | 3 (2.4) | 98 (77.8) | 19 (15.1) | 4 (3.2) | 5 (4.0) |  |
| **Year of diagnosis** | |  |  |  |  |  |  |  |  |  |  |  |  |  |  |  |  |  |
| 1994-1997 | 843 (36.3) | 760 (90.2) | 43 (5.1) | 30 (3.6) | 10 (1.2)^§§^ | 714 (84.7) | 82 (9.7) | 24 (2.9) | 23 (2.7)^NS^ | 727 (86.2) | 56 (6.6) | 42 (5.0) | 18 (2.1)^NS^ | 668 (79.2) | 110 (13.1) | 34 (4.0) | 31 (3.7)^NS^ |  |
| 1998-2001 | 815 (35.1) | 722 (88.6) | 40 (4.9) | 38 (4.7) | 15 (1.8) | 689 (84.5) | 79 (9.7) | 20 (2.5) | 27 (3.3) | 677 (83.1) | 62 (7.6) | 41 (5.0) | 35 (4.3) | 634 (77.8) | 96 (11.8) | 38 (4.7) | 47 (5.8) |  |
| 2002-2005 | 665 (28.6) | 595 (89.5) | 22 (3.3) | 26 (3.9) | 22 (3.3) | 557 (83.8) | 62 (9.3) | 19 (2.9) | 27 (4.1) | 558 (83.9) | 40 (6.0) | 35 (5.3) | 32 (4.8) | 503 (75.6) | 86 (12.9) | 35 (5.3) | 41 (6.2) |  |

P values from Pearson’s chi-squared test

^§^ p=0.02 * p=0.002 ^‡^ p=0.006 ^NS^ Not significant at 5%

^§§^ p=0.04 ** p=0.004 ^$^ p=0.0001

**Table S2: Mortality by comorbidity measures**

| **Comorbidity** | **Mortality** | |  |  |  |
| --- | --- | --- | --- | --- | --- |
|  | **One-year look-back period** | **Five-year look-back period** |  |  |  |
|  | **HR (95%CI)^a^** | **HR (95%CI)^a^** |  |  |  |
| **Cervical cancer** |  |  |  |  |  |
| Charlson (1 unit) | 1.28 (1.14-1.44) | 1.21 (1.09-1.35) |  |  |  |
| Charlson 0 | 1.00^b^ | 1.00^b^ |  |  |  |
| Charlson 1 | 1.41 (0.93-2.13) | 1.17 (0.81-1.70) |  |  |  |
| Charlson 2 | 1.70 (1.14-2.55) | 1.58 (1.09-2.30) |  |  |  |
| Charlson 3+ | 3.22 (1.73-5.99) | 2.06 (1.25-3.42) |  |  |  |
| Elixhauser (1 unit) | 1.25 (1.11-1.40) | 1.18 (1.07-1.30) |  |  |  |
| Elixhauser 0 | 1.00^b^ | 1.00^b^ |  |  |  |
| Elixhauser 1 | 1.29 (0.96-1.75) | 1.29 (0.98-1.71) |  |  |  |
| Elixhauser 2 | 1.33 (0.83-2.13) | 1.39 (0.92-2.10) |  |  |  |
| Elixhauser 3+ | 2.17 (1.32-3.56) | 1.66 (1.07-2.60) |  |  |  |
| **Other mortality (not cervical cancer)** | |  |  | | |
| Charlson (1 unit) | 1.64 (1.35-2.00) | 1.69 (1.43-1.98) |  | | |
| Charlson 0 | 1.00^b^ | 1.00^b^ |  |  |  |
| Charlson 1 | 1.35 (0.57-3.19) | 2.65 (1.44-4.86) |  |  |  |
| Charlson 2 | 4.21 (2.08-8.51) | 5.54 (2.85-10.78) |  |  |  |
| Charlson 3+ | 5.18 (1.57-17.04) | 6.30 (2.71-14.65) |  |  |  |
| Elixhauser (1 unit) | 1.46 (1.18-1.79) | 1.64 (1.41-1.91) |  |  |  |
| Elixhauser 0 | 1.00^b^ | 1.00^b^ |  |  |  |
| Elixhauser 1 | 2.49 (1.39-4.44) | 2.51 (1.39-4.53) |  |  |  |
| Elixhauser 2 | 2.62 (1.20-5.72) | 3.66 (1.80-7.44) |  |  |  |
| Elixhauser 3+ | 2.76 (1.04-7.30) | 7.29 (3.71-14.29) |  |  |  |
| **Total mortality** |  |  |  |  |  |
| Charlson (1 unit) | 1.34 (1.21-1.48) | 1.30 (1.19-1.42) |  |  |  |
| Charlson 0 | 1.00^b^ | 1.00^b^ |  |  |  |
| Charlson 1 | 1.38 (0.95-2.01) | 1.41 (1.03-1.94) |  |  |  |
| Charlson 2 | 2.10 (1.49-2.95) | 2.01 (1.46-2.76) |  |  |  |
| Charlson 3+ | 3.40 (1.96-5.91) | 2.49 (1.62-3.83) |  |  |  |
| Elixhauser (1 unit) | 1.28 (1.15-1.41) | 1.26 (1.16-1.36) |  |  |  |
| Elixhauser 0 | 1.00^b^ | 1.00^b^ |  |  |  |
| Elixhauser 1 | 1.47 (1.13-1.92) | 1.46 (1.14-1.87) |  |  |  |
| Elixhauser 2 | 1.48 (0.99-2.21) | 1.66 (1.17-2.37) |  |  |  |
| Elixhauser 3+ | 2.20 (1.41-3.41) | 2.23 (1.55-3.20) |  |  |  |
|  |  |  |  |  |  |
| ^a^ Adjusted for age, year of diagnosis, stage, ethnicity, NZDep, urban/rural residence | | | |  |  |
| ^b^ Reference category |  |  |  |  |  |

**Table S3: Elixhauser comorbid conditions frequency and cervical cancer-specific mortality adjusted for individual comorbid conditions**

| **Comorbidity** | **Frequency, n (%)** | |  | **HR (95%CI)*** | |  |
| --- | --- | --- | --- | --- | --- | --- |
|  | **One-year look-back period** | **Five-year look-back period** |  | **One-year look-back period** | **Five-year look-back period** |  |
| Congestive heart failure | 33 (1.4) | 51 (2.2) |  | 2.35 (1.22-4.52) | 1.76 (1.01-3.08) |  |
| Cardiac arrhythmia | 35 (1.5) | 54 (2.3) |  | 1.38 (0.60-3.18) | 1.08 (0.54-2.13) |  |
| Valvular disease | 8 (0.3) | 16 (0.7) |  | 2.84 (0.70-11.61) | 1.41 (0.35-5.71) |  |
| Pulmonary circulation disorders | 6 (0.3) | 12 (0.5) |  | - | 1.54 (0.38-6.27) |  |
| Peripheral vascular disorders | 14 (0.6) | 25 (1.1) |  | 1.15 (0.36-3.61) | 0.98 (0.36-2.67) |  |
| Hypertension uncomplicated | 104 (4.5) | 143 (6.2) |  | 0.98 (0.63-1.52) | 1.02 (0.69-1.51) |  |
| Hypertension complicated | 4 (0.2) | 5 (0.2) |  | 1.74 (0.24-12.72) | 1.74 (0.24-12.72) |  |
| Paralysis | 17 (0.7) | 29 (1.3) |  | 1.26 (0.40-3.99) | 0.94 (0.39-2.30) |  |
| Other neurological disorders | 20 (0.9) | 31 (1.3) |  | 1.22 (0.30-4.99) | 1.30 (0.47-3.55) |  |
| Chronic pulmonary disease | 56 (2.4) | 96 (4.1) |  | 1.62 (0.95-2.77) | 1.34 (0.85-2.11) |  |
| Diabetes uncomplicated | 57 (2.5) | 70 (3.0) |  | 2.17 (1.33-3.53) | 2.07 (1.32-3.27) |  |
| Diabetes complicated | 15 (0.7) | 21 (0.9) |  | 10.46 (3.01-36.37) | 10.46 (3.01-36.37) |  |
| Hypothyroidism | 12 (0.5) | 18 (0.8) |  | 0.31 (0.07-1.27) | 0.41 (0.13-1.33) |  |
| Renal failure | 27 (1.2) | 32 (1.4) |  | 4.27 (2.08-8.76) | 3.71 (1.83-7.50) |  |
| Liver disease | 13 (0.6) | 21 (0.9) |  | 2.43 (0.76-7.78) | 1.39 (0.44-4.38) |  |
| Peptic ulcer disease excluding bleeding | 3 (0.1) | 6 (0.3) |  | - | - |  |
| AIDS/HIV | 0 | 0 |  | - | - |  |
| Lymphoma | 2 (0.1) | 4 (0.2) |  | 0.90 (0.12-6.60) | 1.03 (0.25-4.24) |  |
| Solid tumour without metastasis | 66 (2.8) | 93 (4.0) |  | 1.15 (0.66-1.99) | 1.12 (0.70-1.81) |  |
| Rheumatoid arthritis/collagen vascular diseases | 7 (0.3) | 13 (0.6) |  | 1.15 (0.42-3.16) | 1.25 (0.55-2.83) |  |
| Coagulopathy | 9 (0.4) | 11 (0.5) |  | 2.78 (0.68-11.43) | 3.61 (1.13-11.53) |  |
| Obesity | 24 (1.0) | 32 (1.4) |  | 3.52 (1.55-7.98) | 3.66 (1.79-7.46) |  |
| Weight loss | 7 (0.3) | 10 (0.4) |  | 0.76 (0.10-5.57) | 0.35 (0.05-2.56) |  |
| Fluid and electrolyte disorders | 34 (1.5) | 54 (2.3) |  | 4.03 (2.01-8.08) | 4.05 (2.25-7.26) |  |
| Blood loss anaemia | 36 (1.6) | 38 (1.6) |  | 2.44 (1.48-4.00) | 2.44 (1.50-3.96) |  |
| Deficiency anaemia | 22 (1.0) | 40 (1.7) |  | 0.57 (0.21-1.55) | 0.83 (0.41-1.69) |  |
| Alcohol abuse | 8 (0.3) | 24 (1.0) |  | 1.23 (0.17-8.95) | 0.43 (0.10-1.82) |  |
| Drug abuse | 4 (0.2) | 10 (0.4) |  | 3.28 (0.45-23.76) | 4.94 (1.21-20.17) |  |
| Psychoses | 7 (0.3) | 21 (0.9) |  | 0.70 (0.10-5.01) | 1.51 (0.56-4.10) |  |
| Depression | 9 (0.4) | 28 (1.2) |  | 1.01 (0.25-4.09) | 1.43 (0.63-3.25) |  |
| * Adjusted for age, year of diagnosis, stage, ethnicity, NZDep, and urban/rural residence | | | | | |  |

For the hazard ratio estimate for each comorbidity the reference group is women that do not have that comorbidity.

| **Table S4: Cervical cancer-specific mortality by ethnicity adjusted for comorbidity with one-year look-back period** | | | | | | |
| --- | --- | --- | --- | --- | --- | --- |
|  | | | | |  |  |
| **Comorbidity** | **Comorbidity** |  | **Ethnicity** | | | |
|  |  |  | **Other** | **Māori** | **Pacific** | **Asian** |
|  | **HR (95%CI)^a^** |  | **HR (95%CI)^b^** | **HR (95%CI)^c^** | **HR (95%CI)^c^** | **HR (95%CI)^c^** |
| No comorbidity adjustment/inclusion |  |  | 1.00 | 1.56 (1.19-2.05) | 1.95 (1.21-3.13) | 0.72 (0.41-1.27) |
| **Indices as continuous variable** |  |  |  |  |  |  |
| Charlson | 1.28 (1.14-1.44) |  | 1.00 | 1.57 (1.20-2.06) | 1.85 (1.15-2.97) | 0.73 (0.42-1.30) |
| Elixhauser | 1.25 (1.11-1.40) |  | 1.00 | 1.55 (1.19-2.04) | 1.92 (1.20-3.09) | 0.72 (0.41-1.26) |
| **Individual comorbid conditions** |  |  |  |  |  |  |
| Congestive heart failure | 2.35 (1.22-4.52) |  | 1.00 | 1.57 (1.20-2.06) | 1.98 (1.23-3.17) | 0.72 (0.41-1.27) |
| Valvular disease | 2.84 (0.70-11.61) |  | 1.00 | 1.56 (1.19-2.04) | 1.96 (1.22-3.14) | 0.72 (0.41-1.27) |
| Hypertension, complicated | 1.74 (0.24-12.72) |  | 1.00 | 1.57 (1.19-2.06) | 1.95 (1.22-3.13) | 0.72 (0.41-1.27) |
| Chronic pulmonary disease | 1.62 (0.95-2.77) |  | 1.00 | 1.55 (1.18-2.03) | 1.95 (1.22-3.13) | 0.67 (0.38-1.19) |
| Diabetes, complicated | 10.46 (3.01-36.37) |  | 1.00 | 1.55 (1.18-2.04) | 1.70 (1.03-2.80) | 0.71 (0.40-1.25) |
| Renal failure | 4.27 (2.08-8.76) |  | 1.00 | 1.58 (1.20-2.07) | 1.70 (1.04-2.77) | 0.72 (0.41-1.27) |
| Liver disease | 2.43 (0.76-7.78) |  | 1.00 | 1.55 (1.18-2.03) | 1.92 (1.20-3.09) | 0.72 (0.41-1.26) |
| Coagulopathy | 2.78 (0.68-11.43) |  | 1.00 | 1.55 (1.18-2.03) | 1.91 (1.19-3.07) | 0.72 (0.41-1.27) |
| Obesity | 3.52 (1.55-7.98) |  | 1.00 | 1.55 (1.18-2.04) | 1.90 (1.18-3.05) | 0.72 (0.41-1.27) |
| Fluid and electrolyte disorders | 4.03 (2.01-8.08) |  | 1.00 | 1.51 (1.15-1.98) | 1.97 (1.23-3.16) | 0.69 (0.39-1.21) |
| Blood loss anaemia | 2.44 (1.48-4.00) |  | 1.00 | 1.53 (1.17-2.01) | 1.98 (1.23-3.17) | 0.71 (0.40-1.26) |
| Drug abuse | 3.28 (0.45-23.76) |  | 1.00 | 1.56 (1.19-2.04) | 1.95 (1.22-3.13) | 0.72 (0.41-1.27) |
| **All 12 of the above** |  |  | 1.00 | 1.44 (1.09-1.89) | 1.62 (0.98-2.68) | 0.63 (0.35-1.13) |
|  |  |  |  |  |  |  |
| ^a^ Adjusted for age, year of diagnosis, stage, ethnicity, NZDep, urban/rural residence | | | | |  |  |
| ^b^ Reference category |  |  |  |  |  |  |
| ^c^ Adjusted for age, year of diagnosis, stage, ethnicity, NZDep, urban/rural residence and comorbidity index | | | | | |  |
